# A69 UVB SKIN EXPOSURE MODULATES INTESTINAL METABOLISM AND HOST DEFENSE

**DOI:** 10.1093/jcag/gwae059.069

**Published:** 2025-02-10

**Authors:** M C Jimenez Sanchez, H Yang, H Sham, J Dutz, B vallance

**Affiliations:** Department of Medicine, The University of British Columbia, Vancouver, BC, Canada; Department of Medicine, The University of British Columbia, Vancouver, BC, Canada; Department of Medicine, The University of British Columbia, Vancouver, BC, Canada; Department of Medicine, The University of British Columbia, Vancouver, BC, Canada; Department of Medicine, The University of British Columbia, Vancouver, BC, Canada

## Abstract

**Background:**

Gastrointestinal (GI) diseases are rising in Western societies, alongside environmental changes like reduced exposure to ultraviolet (UV) B light. UVB light affects the skin through vitamin D (vitD) and Aryl hydrocarbon receptor (Ahr) signaling, but its potential influence on distant sites, like the gut, is not well studied, despite GI and skin diseases often co-occurring (O’Neill et al, 2016). Patients not only suffer from GI symptoms but also may experience various mood disorders such as anxiety and depression (Graff et al, 2009). We aimed to explore how UVB skin exposure influences gut health, microbiota and its metabolic by-products, and hypothesize that UVB may also affect the gut-brain axis, influencing mood and cognition.

**Aims:**

Test the (1) effect of UVB light using vitD deficient (-) and sufficient (+) diets on the metabolomic profile and gut microbiome, and (2) the impact of vitD levels and UVB on susceptibility to *Citrobacter rodentium* infection.

**Methods:**

Female C57BL/6J mice were fed either vitD (+) or (-) diets. They were anaesthetized, and then 8 cm^2^ of their dorsal skin was shaved and exposed to UVB. Mice were euthanized to collect serum, cecal content, and stool (FI-ToF metabolomics analysis and 16S sequencing). Ingenuity Pathway Analysis (IPA) was used to predict the impact of metabolic changes.

Mice were infected by oral gavage using 2x10^8^ colony-forming units of WT C. rodentium, monitored daily and euthanized on day 10 post-infection. Samples (cecum, colon, and stool) were collected for CFU, histology, immunostaining, and qPCR analysis.

**Results:**

In vitD (-) fed mice, UVB skin exposure raised vitD levels to match those on vitD (+) diets. Untargeted metabolomics detected ~1,630 stool metabolites at baseline, and following one UVB exposure, 266 metabolites changed in the vitD (-) group, 297 in the vitD (+), with 47 overlapping. After 6 exposures, 449 metabolites changed in the vitD (-) group, 138 in the vit D (+), with 61 overlapping. Pathway analysis revealed significant alterations in purine, tryptophan, and alanine-aspartate-glutamate metabolism. IPA determined that changes in these pathways in the vitD (-) group were associated with mood disorders and colorectal tumor inhibition. 16S microbiome analysis showed changes in the Bacteroidota phylum. Finally, vitD (-) group showed increased susceptibility to *C. rodentium* infection, while UVB light skin exposure reduced intestinal pathology in the vitD (-) group, suggesting a protective role of UVB light against intestinal infection.

**Conclusions:**

The study demonstrates that UVB exposure significantly alters the GI metabolome and microbiome, with a greater impact in vitD (-) mice. These findings highlight the complex interplay between UVB exposure, vitD status, and host metabolism and immunity.

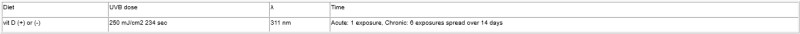

**Funding Agencies:**

NSERC

